# Improved CRISPR/Cas9 gene editing by fluorescence activated cell sorting of green fluorescence protein tagged protoplasts

**DOI:** 10.1186/s12896-019-0530-x

**Published:** 2019-06-17

**Authors:** Bent Larsen Petersen, Svenning Rune Möller, Jozef Mravec, Bodil Jørgensen, Mikkel Christensen, Ying Liu, Hans H. Wandall, Eric Paul Bennett, Zhang Yang

**Affiliations:** 1 Department of Plant and Environmental Sciences, University of Copenhagen, DK-1871 Frederiksberg C, Denmark; 2 Present Address: Centre for Novel Agricultural Products, University of York, Woodsmill Quay, Skeldergate, York, YO1 6DX UK; 3 Present Address: UIT - Department of Chemistry, The Arctic University of Norway, Forskningsparken. 3, 9019 Tromsø, Norway; 4 Copenhagen Center for Glycomics, Department of Molecular and Cellular Medicine and School of Dentistry, Faculty of Health Sciences, University of Copenhagen, DK-2200 Copenhagen N, Denmark

**Keywords:** Precise genetic editing, Genome engineering, CRISPR/Cas9, Protoplasting, Fluorescence activated cell sorting, Mutation enrichment, *Nicotiana benthamiana*

## Abstract

**Background:**

CRISPR/Cas9 is widely used for precise genetic editing in various organisms. CRISPR/Cas9 editing may in many plants be hampered by the presence of complex and high ploidy genomes and inefficient or poorly controlled delivery of the CRISPR/Cas9 components to gamete cells or cells with regenerative potential. Optimized strategies and methods to overcome these challenges are therefore in demand.

**Results:**

In this study we investigated the feasibility of improving CRISPR/Cas9 editing efficiency by Fluorescence Activated Cell Sorting (FACS) of protoplasts. We used *Agrobacterium* infiltration in leaves of *Nicotiana benthamiana* for delivery of viral replicons for high level expression of gRNAs designed to target two loci in the genome, *NbPDS* and *NbRRA*, together with the Cas9 nuclease in fusion with the 2A self-splicing sequence and GFP (Cas9-2A-GFP). Protoplasts isolated from the infiltrated leaves were then subjected to FACS for selection of GFP enriched protoplast populations. This procedure resulted in a 3–5 fold (from 20 to 30% in unsorted to more than 80% in sorted) increase in mutation frequencies as evidenced by restriction enzyme analysis and the Indel Detection by Amplicon Analysis, which allows for high throughput profiling and quantification of the generated mutations.

**Conclusions:**

FACS of protoplasts expressing GFP tagged CRISPR/Cas9, delivered through *A. tumefaciens* leaf infiltration, facilitated clear CRISPR/Cas9 mediated mutation enrichment in selected protoplast populations.

**Electronic supplementary material:**

The online version of this article (10.1186/s12896-019-0530-x) contains supplementary material, which is available to authorized users.

## Background

CRISPR/Cas has emerged as a powerful tool for precise genetic editing (PGE) in a wide range of organisms [1], including plants [2]. CRISPR/Cas relies on the Cas DNA nuclease being guided by the small guide RNA (gRNA), to make a double stranded break (DSB) at the desired place in the genome (reviewed in [3]) leading to activation of inherent repair mechanisms (Non-Homologous End Joining (NHEJ) or Homologous Recombination (HR) if a DNA molecule with identical flanking sequences is co-delivered. CRISPR/Cas mediated PGE in plants may be complicated by the presence of complex and high ploidy genomes or by inefficient or poorly controlled delivery of PGE components to gamete cells or cells with regenerative potential. Moreover, subsequent regeneration and tissue culturing post PGE is often lengthy, labor-intensive and prone to produce random somatic mutations and targeted insertion mediated mutagenesis through homologous recombination is still a main challenge within PGE [2]. There is therefore a demand for optimizing PGE in plants towards efficient generation and propagation of stable heritable editing at the organism level.

Nucleic acids may be introduced into plant cells/tissues by biolistic particle bombardment [4], which, however, often results in insertion of multiple copies at multiple sites in the genome [5]. Other strategies include transformation of protoplast by chemical means using polyethylene glycol (PEG) in combination with calcium ions or by electroporation (reviewed in [5]), where the latter requires elaborate tissue culturing for regeneration to fertile plants and may introduce genetic instability and resulting somaclonal variation. PEG-mediated transformation, in particular, has been used to deliver constructs encoding the PGE components, incl. Zinc Finger-Nucleases (ZFNs) [6], Transcription activator-like effector nucleases (TALENs) [7, 8] and CRISPR/Cas9 [8, 9] and lately also for delivery of the Cas9 enzyme and associated gRNA into plant cell protoplasts in vitro [10]. Excess DNA is regularly used for PEG mediated transformation of protoplasts (typically in molar ratios of 1: 1–2 × 10^7^ (protoplast: plasmid DNA) [11]) and has been reported to confer unintended random integrations in the recipient genomes [12]. *Agrobacterium*-mediated transformation on the other hand is generally perceived to be an efficient and a more controlled way of delivering transgenes [13] and the use of strains, with putatively downregulated integration capacity [14], in combination with down-regulation of host factor integration genes may facilitate alternative ways of non-integrative delivery of PGE components. Also, *Agrobacterium* may in some cases be the only viable option for delivering of transgenes. In recent years, *Agrobacterium*-mediated delivered viral constructs has attracted increasing interest because of their high copy number and resulting expression capabilities [15, 16]. Deconstructed viral vectors (replicons) have proved extremely effective for rapid, high-yield production of a number of pharmaceutical proteins, of which some are currently undergoing clinical evaluation [16]. As efficient gene editing relies on PGE component expression, virus replicons have likewise attracted attention as delivery vehicles [17]. Deconstructed geminivirus type replicons (as delivery vehicles) have been shown to generate mutations in the solanaceous species *Nicotiana benthamiana* [17] and *Solanum lycopersicum* (tomato) [18] and recently in *Triticum aestivum* (wheat) [19]. *N. benthamiana* can be grown in high density and still produce large amounts of biomass in a matter of weeks [16], and has a track record for production of therapeutic glycoproteins in mg scale ([20–24]) through the use of leaf or leaf disc infiltration [25]. In addition, *N. benthamiana* may readily be subjected to protoplast transformation [26] and explant/protoplast regeneration [27, 28]. Several approaches have been reported to confer enrichment of PGE mutations in cells. Fluorescence Activated Cell Sorting (FACS) of edited cells, for example, is regularly used as means of PGE mutation enrichment in mammalian cell systems [29], and the present study addresses the feasibility of applying this strategy to plant cells.

So far reports on FACS and post FACS cultivation of plant protoplasts are relatively scarce [30], due to the removal of the rigid and structure providing cell wall, which otherwise stabilize the cell integrity [31–33]. The present study explores the combined use of *Agrobacterium*-mediated delivery of viral replicons for expression of GFP tagged gRNA/Cas9 in leaves of *N. benthamiana* with FACS in order to obtain protoplast populations with significantly increased gene editing.

## Results

**Fig. 1:**
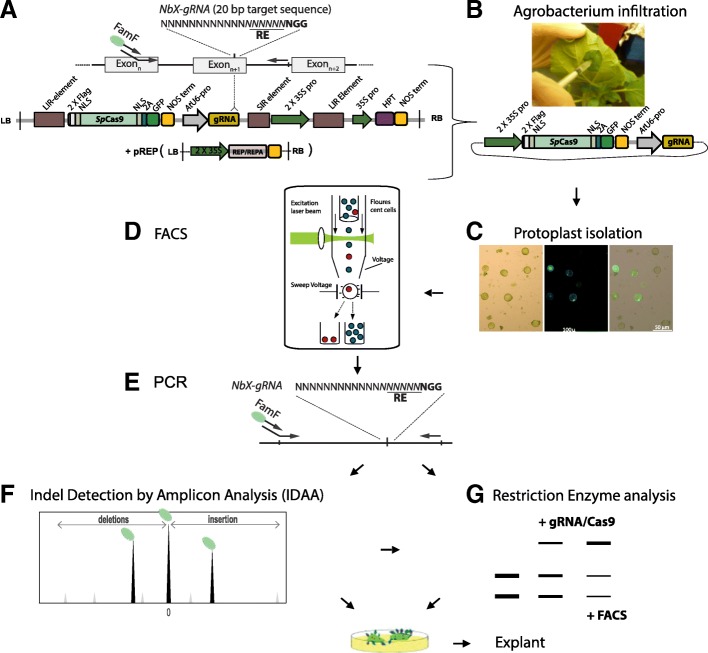
*Scheme for Agrobacterium-mediated in-leaf GFP tagged CRISPR/Cas9 mutation generation combined with FACS enrichment of GFP expressing protoplasts*. **a** Guide RNA (gRNA) target sequence may be selected on the basis of in silico prediction analysis and the presence of a Restriction Enzyme (RE) recognition motif spanning the *Sp*Cas9 cleavage site (− 3 bp upstream of the protospacer adjacent motif (PAM) [34]) for fast RE-mediated mutation screening. Primers flanking the gRNA target for PCR mediated-mutation scoring are indicated. The deconstructed bean yellow dwarf virus (*BeYDV*) replicon is produced from the *Agrobacterium tumefaciens* delivered T-DNA, that contains the viral *cis*-acting Long (LIR) and Short Intergenic Regions (SIR) in a Long-Short-Long region (pLSL) arrangement, which together with the co-expressed *trans* acting Rep/RepA replication initiation proteins facilitate replicational release and Gemini Virus Replicon (GVR) circularization allowing joining of the two *BeYDV* replicon LIR elements within plant cell nuclei [17]. Abbreviations: Left and Right T-DNA border, LB & RB, *Cauliflower mosaic virus 35S* promoter, *CMV35S*, *Arabidopsis thaliana U6* promotor, *AtU6*-Pro [35, 36], hygromycin phosphotransferase, HPT, *Streptococcus pyogenes* Cas9, *Sp*Cas9, nopaline synthase terminator, NOS, Nucleus Localization Signal, NLS, 2A self-cleaving sequence of foot-and-mouth disease virus (FMDV), 2A [37, 38], *Agrobacterium tumefaciens, A. tumefaciens, Nicotiana benthamiana, N. benthamiana*. The replicon constructs (**a**) are transformed into *A. tumefaciens* by electroporation, grown under selection overnight and re-suspended in infiltration buffer to a final total OD_600_ of ca. 0.2 where after the abaxial side of young expanding leaves of 3–4 week old *N. benthamiana* plants are infiltrated with the agrobacterium strain carrying the construct of interest using a syringe and left for 2–4 days (**b**). Protoplasts are isolated (**c**) and subjected to florescence microscopy (for estimation of protoplast isolation and transformation efficiencies) and to Fluorescence Activated Cell Sorting (FACS) (**d**) of GFP (*Sp*Cas9-2A-GFP) expressing protoplasts for mutation enrichment. The target region on the genome is amplified by PCR (**e**) with mutations scored by the high throughput screening technique Indel Detection by Amplicon Analysis (IDAA) [39] (**f**), which allows for detection of down to 1 bp deletions and insertions (indels) and by restriction enzyme (RE) analysis (**g**), which monitors resistant mutated RE recognition/cleavage sites. Optionally, explants with stable PGE editing can be obtained by embedding the protoplasts in alginate, followed by callus induction and shoot regeneration as outlined in [40]. Protoplasts shown in (**c**) are presented as light-, fluorescent micrographs and overlay hereof

The overall strategy for *Agrobacterium-*mediated delivery of deconstructed replicons expressing gRNA/Cas9-2A-GFP in leaves of *N. benthamiana* combined with FACS of GFP expressing protoplasts is outlined in Fig. 1.

### gRNA and replicon construct design

**Fig. 2:**
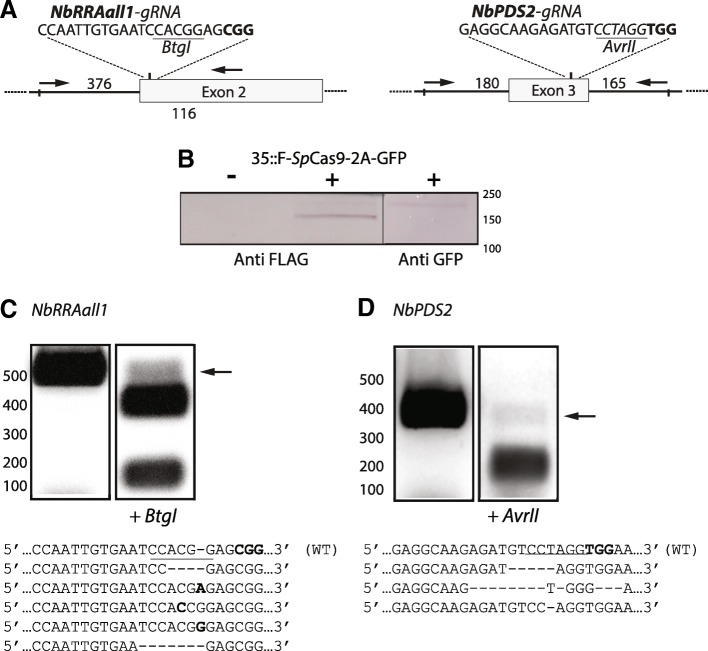
*NbRRA*all*1/NbPDS2-gRNA generated indels*. **a** gRNA targets of the *N. benthamiana* loci, *REDUCED RESIDUAL ARABINOSE* arabinosyl transferase (*NbRRA*) and *PHYTOENE DESATURASE* (*NbPDS*), were a *Btg*I and a *Avr*II site is situated 2 and 0 bp upstream of the protospacer adjacent motif (PAM), respectively. Given the predicted cut site of *Sp*Cas9, 3 bp upstream of the PAM sequence [44], all of the *NbPDS2*-gRNA derived mutation combinations will destroy the *Avr*II site in the *NbPDS* target site and only insertions starting with ‘G’ at the cut site, i.e. less than one fourth the insertions possible, will restore the *Btg*I site in the *NbRRA*all*1* target site. Primers, flanking the gRNA targets, are indicated by arrows. **b** Western blot analysis of day 4 post infiltration leaves using anti Flag and anti GFP mAbs, cross-reacting to *Sp*Cas9 (154 kDa) and to a faint protein band corresponding to the un-cleaved fusion protein (*Sp*Cas9-2A-GFP, ca 180 kDa), respectively. **c, d** DNA from 4 days post infiltration leaf samples of *NbRRA*all1- and *NbPDS2*-gRNA/Cas9 infiltrations were isolated, PCR amplified and subjected to restriction enzyme digestions using *Btg*I (*NbRRA*all*1*) and *Avr*II (*NbPDS2*), respectively, with the resistant bands (indicated by arrow) isolated, cloned into pJet and 12 clones of each target sequenced revealing the resulting indels depicted

In the present study we targeted the *Nicotiana benthamiana PHYTOENE DESATURASE* (*NbPDS*) and *REDUCED RESIDUAL ARABINOSE* arabinosyl transferase (*NbRRA*) loci, orthologous to the *Arabidopsis thaliana* arabinosyltransferase encoding genes involved arabinosylation of plant cell wall extensins (*AtRRA1–3*) [41, 42], which have a proven [43] and an untested CRISPR/Cas9 editing record, respectively (Fig. 2a). gRNA target sequences were confined to early exons and identified on the basis of in silico prediction analysis (http://portals.broadinstitute.org/gpp/public/analysis-tools/sgrna-design, [45]), and the presence of a Restriction Enzyme (RE) recognition sequence spanning the predicted cut site of *Sp*Cas9–3 bp upstream of the protospacer adjacent motif (PAM) [34] for RE-mediated mutation screening.

A deconstructed immobilized mild strain of the bean yellow dwarf virus (*BeYDV*), allowing for a high replicon copy number in the nucleus, has recently been used to construct an *Agrobacterium* T-DNA that integrates into the host cell chromosome and delivers a geminivirus replicon (GVR) [17, 46]. The minimal immobilized replicons are delivered by *Agrobacterium* infiltration (here to *N. benthamiana* leaves) along with co-infiltrated constructs for expression of replicon trans-acting replication initiation proteins (Rep or RepA) [47] (Fig. 1a). While the replicons are non-integrative and transiently expressed the initial *Agrobacterium* T-DNA (LB-RB) delivery of the replicon is integrative [17]. Lately, GVRs were constructed and used to propagate and express PGE components, such as ZFNs and TALENs and CRISPR/Cas9 [17]. In the present study, we inserted the *Streptococcus pyogenes* Cas9 enzyme (*Sp*Cas9) [48] in translational fusion with the 2A self-splicing sequence of the foot-and-mouth disease virus [37, 38] and GFP [49] (*Sp*Cas9-2A-GFP) under control of the CMV 35S promoter and the gRNAs under control of the *AtU6* promoter [35, 36] in the *BeYDV* GVR replicon [17] as depicted in Fig. 1a and detailed in the Methods section.

### In-leaf gRNA/Cas9 generated mutations

The *Sp*Cas9-2A-GFP/gRNA expressing GVR replicons (Fig. 1a) targeting the *NbPDS* and *NbRRA* loci (Fig. 2a) were electroporated into *Agrobacterium tumefaciens* and grown under selection overnight and re-suspended in infiltration buffer to a final total OD_600_ of 0.2 where after the abaxial sides of young expanding leaves of *N. benthamiana* were subjected to *Agrobacterium* infiltration. The infiltrated plants were left for 2–4 days allowing for gRNA/Cas9 expression and mutation generation within the intact leaves. Western blot analysis of total proteinacious extracts, using anti-Flag and anti-GFP mAbs as primary antibodies against the Flag- and GFP-tagged *Sp*Cas9 revealed the presence of a distinct band at the expected MW (154 kDa) of mature *Sp*Cas9 with a faint band corresponding to the un-cleaved fusion protein (*Sp*Cas9-2A-GFP, ca 180 kDa) in infiltrated leaves demonstrating expression and efficient 2A mediated auto cleavage of *Sp*Cas9-2A-GFP (Fig. 2b).

RE-mediated mutation analysis of PCR fragments, using primers flanking the gRNA target sites, revealed the presence of non-digestible bands indicative of mutated RE recognition/cleavage sequence for the two target sites (Fig. 2c and d). The RE resistant band of each locus was isolated, sub-cloned and sequenced with the presence of insertions or deletions (indels) demonstrated (Fig. 2c and d).

### Protoplast isolation and FACS-mediated mutation enrichment

Protoplasts of WT and infiltrated leaves were essentially obtained using the protocol devised by Dovzhenko et al. 1998 [27] with minor modifications as outlined in the Methods section. Protoplast quality and yield varied significantly apparently influenced by growth conditions pre- and post-infiltration. Here a temperature of 22–24 °C, a 16 h/8 h (light/dark) regime of moderate sunlight (See ‘Growth conditions’, Methods section) generally conferred a high amount of intact protoplasts. Protoplast integrity and transformation were assessed by comparative bright field and fluorescent microscopy frequently with varying estimated transformation rates of 20 - > 80% (Additional file 1: Figure S1). GFP fluorescence accumulated in particular in the cytoplasmic strands and the contours of the cell (Additional file 1: Figure S1), which is in agreement with a cytoplasmic 2A-mediated release of GFP. This was corroborated by the western blot analysis (Fig. 2b) showing presence of the mature *Sp*Cas9 with only traces un-cleaved product. Also, in agreement with soluble non-tagged GFP being able to pass into and accumulate in nuclei [50], some accumulation in nuclei structures was observed (Additional file 1: Figure S1).

**Fig. 3:**
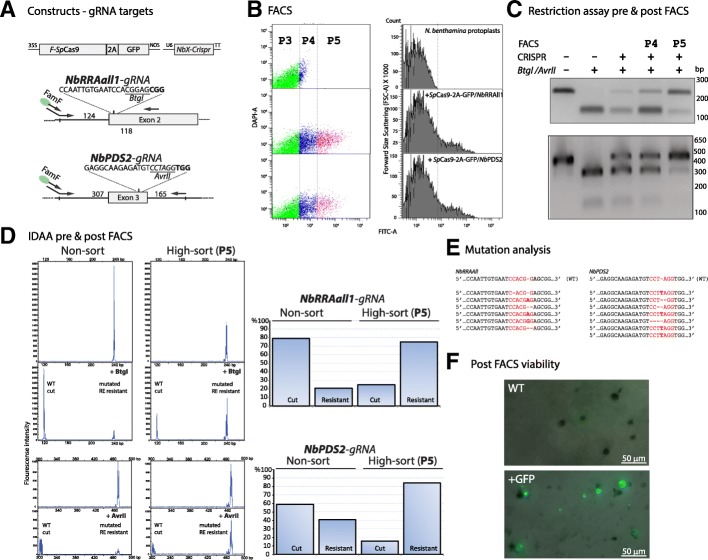
*FACS mediated enrichment of gRNA/SpCas9 expressing protoplast cells and resulting mutations*. 3–5 *N. benthamiana* leaves were infiltrated with *Agrobacterium*-delivered replicons expressing *Sp*Cas9-2A-GFP together with *NbRRA*all-gRNA or *NbPDS2-*gRNA (**a**), respectively, and left for 2–4 days. **b** WT protoplasts and protoplasts expressing *Sp*Cas9-2A-GFP and *NbRRA*all1- or *NbPDS2*-gRNA were subjected to GFP mediated FACS. The DAPI and FITC intensities for protoplasts were recorded and three populations, P3, P4 & P5, with the P3 population corresponding to non-transformed cells and the P4 and P5 populations representing intermediary and high stringently sorted cell populations, respectively, were selected from the Dot Scattering Chromatograms. Transfected protoplasts were defined as FITC-positive events and gates were set to separate WT and GFP enriched protoplast populations, using the WT sample to define non-transfected wild type populations (P3) in the transfected samples. P4 and P5 (GFP enriched populations) were gated with medium and high FITC signal intensity. **c** RE analysis of PCR amplified target regions using *Btg*I and *Avr*II for *NbRRA*all1- and *NbPDS2* gRNAs, respectively, demonstrating indel formation in unsorted and indel enrichment in FACS sorted (P4 and P5) populations. Indel enrichment in P5 populations were cooperated by the IDAA technique (**d**) where the additional restriction enzyme digest allows for visualization of the mutated population without the presence of non-mutated PCR amplicons (‘mutated / RE resistant’ designates the RE site were mutated rendering it resistant for digestion while ‘WT/cut’ designates WT sites that were cut and moved downstream in the chromatogram). **e** Sequence analysis of RE-resistant PCR fragments of the two P5 populations. **f** Post viability of protoplasts was assessed in WT protoplasts (dark circular objects) without detectable GFP signal and GFP fluorescence in Cas9-2A-GFP sorted protoplasts (presented as an overlay of light and fluorescent micrographs). FACS was carried out using a FACSAria III (BD Biosciences) apparatus with procedure and parameters as outlined in the Methods section and IDDA as described in [39]. For the viability test shown in **f** crude protoplasts were prepared and sorted on a Sony Cell sorter SH800S with sorting gating parameters similar to those used on the BD FACSAria III sorter and with the W5 buffer as recipient buffer

FACS of fluorescent protoplasts were done using a FACSAria III (BD Biosciences) apparatus with settings to accommodate for the approximate size of *N. benthamiana* protoplasts [51] as described in the Methods section. Two fluorescent enriched populations, protoplasts with medium GFP intensity (P4) and with high intensity (P5), were selected for sorting corresponding to 17% & 10 and 14% & 5% of the total population for the *NbRRA*all1-gRNA/*Sp*Cas9-2A-GFP and *NbPDS2*-gRNA/*Sp*Cas9-2A-GFP infiltrations, respectively (Fig. 3b). RE analysis of PCR amplicons suggested an estimated indel frequency of unsorted, P4 and P5 sorted populations of 20–30, 50% and 70–80% for the *NbRRA*all1-gRNA and 40, 50 and > 80% for the *NbPDS2*-gRNA (Fig. 3c). This was corroborated by Indel Detection by Amplicon Analysis (IDAA) (Fig. 3d) and sequence analysis of the cloned PCR fragments of the two P5 populations (10 clones of each), which showed indel to WT ratio of 60 and 70%, respectively. The indel distributions obtained for the *NbRRA*all1- and *NbPDS2*-gRNA infiltrations − 3(1), − 1(2) & + 1(4) and − 1(3) & + 1(3) (Fig. 3e), respectively, are in agreement with earlier findings for *Sp*Cas9 mediated mutations in plants [52].

Bright field microscopy suggested that 10–20% of a WT protoplast population was intact after FACS when using PBS buffer as sheath fluid and MMM550 as recipient buffer (Additional file 2: Figure S2). Viability of post FACS GFP positive protoplasts was assessed by bright field and fluorescence microscopy (Fig. 3f) and confirmed by propidium iodide exclusion assays (Additional file 2: Figure S2). Sorting into PBS as recipient buffer resulted in instant lysis as evidenced by bright field microscopy (data not shown). In agreement with ribonucleoprotein, i.e. in vitro transcribed gRNA and heterologous expressed Cas9, conferring nuclease activity in-vitro [10, 53], we tested (post FACS) for PBS lysis mediated editing activity and found an 2–3 fold increased editing when the PBS lysed protoplasts were left in PBS for 2 h at room temperature (Additional file 3: Figure S3). All post FACS protoplast samples were immediately incubated on ice accordingly. Potential continued editing in the timespan from FACS to further processing may on the other hand likewise increase the ‘in cell’ editing.

Embedment of GFP transformed protoplasts in alginate with initial callus formation (Additional file 4: Figure S4) demonstrated the feasibility of obtaining gene edited lines using an explant shoot regeneration systems as described in [40].

## Discussion

The use of PGE in plants may be complicated by the presence of complex genomes and inefficient or poorly controlled delivery of PGE components to gamete cells or recipient pluripotent cells. DNA encoding PGE components may be delivered to the plant cell either directly, i.e. by biolistic transformation or protoplast transformation (reviewed by [5]), or indirectly, mainly via bacteria, usually *Agrobacterium tumefaciens* or (less commonly) *Agrobacterium rhizogenes* [54], which is generally perceived to be a controlled way of delivering transgenes [55]. Virus replicons provide high copy number of expression units and thus a means of significantly boosting PGE component expression levels [46, 56] and methods for increasing identification/selection of PGE-edited cells have been introduced and applied successfully e.g. for mammal cells [29].

In the present study we combined *Agrobacterium*-mediated delivery of a viral replicon expressing GFP labeled gRNA/*Sp*Cas9 for generation of in-leaf mutations with the use of FACS of GFP-fluorescent protoplasts for enrichment of mutated protoplast populations. *BeYDV* GVR replicons, expressing gRNAs targeting the *NbPDS* and the *NbRRA* locus in *N. benthamiana*, respectively, together with the *Sp*Cas9 nuclease, fused to the 2A self-splicing sequence and GFP (SpCas9-2A-GFP), were introduced into leaves of *N. benthamiana* by *Agrobacterium*-mediated infiltration and left for expression and mutation generation within the intact leaf. In leaf expression of the GFP- and Flag-tagged *Sp*Cas9 enzyme was readily verified by western blot analysis and generated mutations as evidenced by the presence of restriction enzyme (RE) resistant bands of PCR amplicons comprising the mutated recognition site were cooperated by cloning and sequence analysis of the RE resistant bands. Indel distribution was found to be in accordance with earlier studies for *Sp*Cas9 mediated genome editing in plants [52]. With the aim of selecting and concentrating edited cells GFP-expressing protoplasts were isolated and subjected to FACS. The two fluorescence-enriched populations were selected for FACS with the most stringently sorted population yielding a 3–5 fold enrichment in mutations as evidenced by RE-mediated mutation analysis of PCR amplicons and sequence analysis.

The IDAA method allows for fast and direct assessment of indel prevalence and distribution [39]. In the current study, IDAA was combined with RE analysis for visualization of the isolated mutation population, where potential single nucleotide substitutions within the RE recognition site will otherwise co-migrate with the WT peak. While the observed > 50% reduction of FAM-fluorescence signal in IDAA analyses of overnight RE digestions may complicate absolute peak quantification between samples, quantification of the WT peak and indel peak(s) within single samples, provides a means of estimating relative mutation efficacies between samples. The combined use of the RE analysis and the IDAA technique adds an extra analytical layer to the versatile IDAA technique. 10–20% of the protoplasts appeared to be intact in post FACS populations, when PBS and the MMM550 buffer were used as sheath fluid and recipient buffer, respectively. This ratio may, however, be increased by replacing the sheath fluid PBS buffer with a more osmotically favorable buffer and, if feasible on the FACS apparatus used, decrease shearing forces by lowering the psi. Here FACS on a Sony SH3800S cell sorter yielded ample intact protoplasts post FACS probably due to the available 130 μm sorting chip with accordingly lower psi. Isolation of non-ruptured protoplasts through the use of a sucrose gradient significantly aids the identification of protoplast populations with and without GFP-expression. Once an initial delineation of the protoplast populations on the cell sorter has been established, this step may potentially be omitted.

Extracellular gRNA/Cas9 activity from lysed protoplasts, e.g. mediated by FACS sorting, was significant and this residual activity, which may lead to an over estimated indel frequency, was abolished by incubation on ice or FACS sorting into an RNAse containing or protein denaturing buffer.

Also, in this study protoplast yields were generally highly variable. A recent study on *Agrobacterium* infiltration-mediated expression of a reporter in leaves of *N. benthamiana* recommended infiltration of more plants but less leaves and sample more positions on the leaf as opposed to run a high number of technical replicates [57]. In addition, it is conceivable that *Agrobacterium* infection/pathogenesis may affect intact protoplasts yield.

Recently, an unexpected high level of integrations in the recipient genomes associated with PEG-mediated plasmid transformation of protoplasts was reported [12]. Further optimization of the here devised PGE approach may include exploring the use of integration deficient *Agrobacterium* strains [58] or Virus Induced Gene Silencing (VIGS) mediated down-regulation of host plant factors [59] also important for T-DNA integration, as means of non-integrative delivery of the PGE components [60]. The obtained mutation enrichment may facilitate mutation detection e.g. in situations where the activity of a particular gRNA is weak and reduce laborious explant generation and screening steps. Alternatively, the protoplast based PGE system may be used e.g. in promoter-reporter editing test-screens.

## Conclusions

The present study outlines a strategy for enrichment of CRISPR/Cas9 editing in leaf protoplasts. GFP tagged gRNA/Cas9 (gRNA/Cas9-2A-GFP) was delivered by *Agrobacterium*-infiltration to leaves of *N. benthamiana* and protoplasts isolated. Subsequent FACS of GFP expressing protoplasts resulted in several fold mutation enrichment in the selected fluorescence enriched protoplast populations.

## Methods

### Growth conditions

Seeds of wild-type *Nicotiana benthamiana* were sown and grown in soil (Pindstrup substrate number 2) for 4 weeks in greenhouse with a 16/8 h light/dark cycle, app. 70% relative humidity and a day/night temperature cycle of 24 and 17 °C.

2 days prior to infiltration plants were subjected to regular sunlight at a photosynthetic flux of 20–40 μmol photons m^− 2^s^− 1^, Photosynthetic Active Radiation (PAR): 20.5 μE.m^− 2^s^− 1^, Red – Far Red ratio (R:FR): 1,69), 22–24 °C temperature, an app. 16 h/8 h (light/dark) diurnal rhythm and 70% relative humidity, which were also imposed in the post-infiltration period.

### Vectors and construct designs

Descriptive naming of vectors, constructs, primers and primer sequences are provided in Additional file 5: Table S1. The vector pLSLGFP-R (V82), described in [17], containing GFP insert in front of the CMV35S promoter and Gateway destination site in front of CMV 35S promoter-LIR, respectively, was kindly provided by Nicholas Baltes, Michigan University, US. The gRNA Gateway entry vector V26 (pUC57_attL1-*AtU6*:*BbsI*-*BbsI*-tracr-TT_AttL2) was synthesized by Genscript. To obtain insertion of *NbRRA*all1- or *NbPDS2* gRNAs V26 was linearized with *Bbs*I and gRNA targets *NbRRA*all1 and *NbPDS2* were inserted by ligation of the annealed oligonucleotides P042 & P043 and P149 & P150, respectively, yielding V207 (attL1-*AtU6*: *NbPDS2*-tracr-TT_AttL2) and V208 (attL1-*AtU6*: *NbRRA*all1-tracr-TT_AttL2). V207 and V208 were linearized using *Eco*RI and cloned together with the *Streptococcus pyogenes* Cas9 (*Sp*Cas9) fragment [11], which was PCR amplified from HBT-Cas9 (Gift from Jen Sheen, Harvard Medical School) using the primer-set P077 & P212, the GFP-Nos fragment amplified from pLSLGFP. R using the primerset L1 & L2, all together using the In-fusion cloning kit (Clontech), yielding V197 (pUC57_AttL1-*Sp*Cas9-2A-GFP-Nos; *AtU6*-*NbRRA*all1-gRNA-TT.AttL2) and V198 (pUC57_AttL1-*Sp*Cas9-2A-GFP-Nos; *A*t*U6*-*NbPDS2*-gRNA-TT-AttL2). V197 & V198 was gateway cloned using pLSL_v2 as destination vector yielding V199 (pLSL_V2_ LIR-AttB1-*Sp*Cas9-2A-GFP-Nos; *AtU6*-*NbRRA*all1-gRNA-TT-AttB2 SIR-35S-LIR) and V200 (pLSL_V2_ LIR-AttB1-*Sp*Cas9-2A-GFP-Nos; *AtU6*-*NbPDS2*-gRNA-TT-AttB2 SIR-35S-LIR), respectively. V199 & V200 will, when co-expressed with pREP, express *Sp*Cas9 in fusion with the 2A self-splicing sequence of the foot-and-mouth disease virus (FMDV) [37, 38] and GFP [49] (*Sp*Cas9-2A-GFP) under control of the CMV35S promoter.

For GFP expression only V82 (pLSLGFP-R_v2) was used.

### PDS (NbPDS) and RRA (NbRRA) target loci in the N. benthamiana chromosome

*N. benthamiana* genes were obtained from https://solgenomics.net/tools/blast/?db_id=266 [61] based on homology with the *Arabidopsis thaliana* genes. As *N. benthamiana* is allotetraploid both chromosome variations of a gene in the given locus are obtained. In contrast to e.g. the presence of 1 and 3 isogenes of *AtPDS* [62] and *AtRRA* [41, 42] in diploid Arabidopsis, respectively, *NbPDS* and *NbRRA* appear to be single gene loci in allotetraploid *N. benthamiana*.

The *NbRRA* gene SolGenomics: Niben101Scf18348 with exons (33526..33687, 35895..36708 & 36767..37113) and Niben101Scf09172 with exons (260530..260692, 261438..262553) with the *NbRRA*all1-gRNA situated in exon 2 (35905..35924, 261512..261531).

The *NbPDS* gene SolGenomics: Niben101Scf14708 with exons (13814..14036, 14118..14251, 15346..15435, 16328..16386, 16604..16760, 17017.. 17166, 17412..17532, 17695..17909 & 18003..18104) and Niben101Scf01283 with exons (198006..198228, 198317..198449, 199413..199501, 200074..200127, 200369..200501, 200792..200940, 201104..201223, 201388..201601 201694..201796, 202066..202113 & 202983..203028) and with the *NbPDS2*-gRNA situated in Exon 3 (15409..15428, 199476..199495).

### Agrobacterium mediated leaf infiltration and expression in Nicotiana benthamiana

*Agrobacterium tumefaciens* pGV3850, harboring constructs (pREP, p19 and (pLSL_V2_ LIR-AttB1-*Sp*Cas9-2A-GFP-Nos; *AtU6*-*NbRRA*all1-gRNA-TT- AttB2 SIR-35S-LIR (V199) or pLSL_V2_LIR-AttB1-*Sp*Cas9-2A-GFP-Nos; *AtU6*-*NbPDS2*-gRNA-TT-AttB2 SIR-35S-LIR (V200) and empty vector control were inoculated in 5 mL YEP media with kanamycin (50 mg/L) and rifampicillin (50 mg/L) and incubated at 28 °C, 250 rpm for 24 h. Cells were harvested by centrifugation for 20 min at 4000×g and re-suspended in infiltration buffer (10 mM MES (Sigma-Aldrich), 10 mM MgCl_2_ and 10 μM acetosyringone (3′,5′-Dimethoxy-4′-Hydroxyacetophenone, Sigma-Aldrich) to a final OD_600_ of ~ 0.2 and incubated for 3 h at room temperature.

The abaxial side of 3–5 young expanding leaves (4–6 × 6–8 cm (Width, Length)) of *N. benthamiana* was infiltrated with *A. tumesfaciens* pGV3850 containing the various constructs and co-infiltrated with the p19 construct [63] (Final OD_600_ = 0.2) essentially as described by Sainsbury and Lomonossoff (2008) [64], and left for 2–4 days depending on the experimental setting.

### Protoplast isolation

Protoplasts were obtained using the protocol devised by Dovzhenko et al. 1998 [27]. Inoculated *N. benthamiana* leaves for subsequent protoplast-alginate embedment were sterilized by dipping in 96% ethanol and floating in 1.5% hypochlorite solution for 15 min. 3–5 leaves were cut into 0.5–1 mm strips with a scalpel and submerged in 10 ml enzyme solution (400 mM mannitol, 20 mM MES-KOH, pH 5.7, 20 mM KCl, supplemented with 1% Cellulase R10 (w/v) (Duchefa Biochemie, C8001), 0.25% Macerozyme (Duchefa Biochemie, C8002), heated to 55 °C, 10 min, then supplemented with 10 mM CaCl_2_ and 0.1% BSA) and incubated 2–5 h at 26 °C, 100 rpm, then filtered through a 100 μm filter into a 50 ml Falcon tube, centrifuged for 5 min at 100×*g,* where after the supernatant was poured off and the protoplast-containing pellet was re-suspended in 3 ml of 10 mM MgSO_4_, 10 mM MgCl_2_, 10 mM MES-KOH, pH 5.8, buffer, 0.5 M mannitol (MMM550) which was carefully layered on top on 8 ml 0.6 M sucrose cushion and spun down at 100×g, 2 min, at room temperature. Intact protoplasts at the interface were collected and spun down at 100×g for 2 mins then re-suspended in MMM550 –– if used for alginate imbedding this step was repeated three times.

For viability test the protoplast-containing pellet was re-suspended in 5 ml 2.5 mM MES-KOH, pH 5.7, 125 mM CaCl_2_, 154 mM NaCl, 5 mM KCl, 0.5 mM glucose (W5), centrifuged for 5 min at 100×*g*, the supernatant poured off, and the pellet re suspended in 0.5 ml W5 and placed on ice until FACS, which was initiated immediately after the wash step.

### Embedment of GFP-fluorescent protoplast in alginate

Protoplast embedment in alginate was essentially done as described in [27] except the thin alginate layer was formed using the ‘droplet on Ca-agar’ method as described in [65]. Briefly, protoplasts re-suspended in 200 μl MMM550 were mixed with 200 μl alginate solution (MMM550 + 2.8% alginate (low viscosity)). A 300 μl droplet was left on a Ca-Agar plate (0.4 M mannitol, 50 mM CaCl_2_, 1% plant agar (Duchefa 1001.5000)) which was tilted to spread out the droplet, and after 30 min a floating solution (0.4 M mannitol, 50 mM CaCl_2_) was added to the plates to allow for movement of the layer. The layer was taken up by a spatula and moved to small Petri dishes containing F-PCN (described in [8]) .

### gDNA extraction

A single fully infiltrated leaf was thoroughly ground in liquid nitrogen and DNA was extracted using DNeasy Plant Mini Kit (Qiagen).

### PCR of genome target *NbRRA and NbPDS* loci

PCR-amplicons containing the *NbRRA* & *Nb*PDS targets were amplified using nested PCR: First 5 μl of protoplast suspension (obtained as described in ‘*Protoplast isolation’*) was used in a 50 μl PCR reaction using Phire Plant Direct PCR Master Mix (ThermoFisher F160S) with the cycle parameters: 5 min at 98 °C followed by 40 cycles of 10 s at 98 °C, 10 s at (65 °C for *RRA* and 62 °C for *PDS*) and 40 s at 72 °C followed by 7 min at 72 °C using the primers P348 & P232 and P346 & P342 for *NbPDS2* and *NbRRA*all1, respectively. Nested *NbRRA* PCR was performed in a 50 μl reaction using X7 polymerase [66] with 1:100 diluted 1’th PCR reaction as template and the cycle parameters: 5 min at 94 °C followed by 25 cycles of 30 s at 94 °C, 30 s at 58 °C and 30 s at 72 °C followed by 7 min at 72 °C and the primers P319 and P320.

Nested PCR of *NbPDS2* was done in a 25 μl reaction using ClonAMP HiFi master mix 2x (Takara 639,298) with the cycle parameters: 5 min at 98 °C followed by 20 cycles of 30 s at 98 °C, 30 s at 65 °C and temperature dropping 0.5 °C per cycle and 30 s at 72 °C followed by 20 cycles of 30 s at 98 °C, 30 s at 58 °C and 30 s at 72 °C followed by 3 min at 72 °C and the primers P321 and P322.

Primers for scoring in leaf mutations were P321 & P322 (*NbRRA*all1) and P232 & P233 (*NbPDS2*).

### Cloning in pJet and sequencing

10 μl of PCR product was digested ON in a 50 μl reaction with *Btg*I (*NbRRA*all1 amplicon) and *Avr*II (*NbPDS* amplicon). Enzyme Resistant bands were isolated from agarose gels using NucleoSpin® Gel and Monarch® DNA Gel Extraction Kit (New England Biolabs) and cloned into pJet1.2 using CloneJET PCR Cloning Kit #K1232. Sequences were aligned using CLC Workbench.

### Indel detection by amplicon analysis (IDAA) and semi quantification of IDAA peaks

Indel Detection by Amplicon Analysis (IDAA) was done essentially as described in and outlined in [39] and in the Method section ‘*PCR of genome target NbRRA and NbPDS loci*’. Briefly A tri-primer PCR setup which relies of the incorporation of a florescent universal 6-FAM 5′-labelled primer (FamF), with the corresponding non-labeled primer in a 1:10 diluted concentration, was used for FAM labeling of PCR amplicons. PCR amplification of the *NbRRA*all1 and *NbPDS2* regions were done using the ClonAMP HiFi master mix 2x (Takara 639,298) in a 25 μl reaction with the cycle parameters: 5 min at 95 °C followed by 30 cycles of 30 s at 95 °C, 30 s at 58 °C and 30 s at 72 °C followed by 3 min at 72 °C. Primers were P230 & P231 (*NbRRA*all1) and P232 & P233 (*NbPDS2*), where bold designates FAM primer overhang (Additional file 5: Table S1).

Mutation frequencies, as identified by quantification of peak area in IDAA chromatograms, were estimated using the Open Source Software program ImageJ (https://imagej.nih.gov/ij/) from and with areas identified as described (http://www.openwetware.org/wiki/Protein_Quantification_Using_ImageJ).

### Fluorescence microscopy

Fluorescence imaging (presence GFP) was carried out with an epifluorescence microscope Olympus BX41 equipped with a CCD camera (FITC filter for GFP fluorescence and DAPI filter for FDA staining) or a laser scanning confocal microscope Leica SP5 equipped with an Argon (448 nm) and a Argon laser (448 nm).

### Western blot analysis

App. 50 μl of seedling powder, crushed in liquid N_2_, was boiled in 50 μl 2 × SDS-PAGE loading buffer (280 mM SDS, 400 mM Tris, 40% glycerol, 1.4 M mercaptoethanol, 0.6 mM Bromophenol Blue) for 15 min and separated (200 V, 50 min) on 12% Criterion XT Bis-Tris gels (Bio-rad). Proteins were electrotransferred onto polyvinylidene difluoride (PVDF) membranes (Bio-rad) using a Trans-Blot® TurboTM Blotting instrument (Bio-rad). The membrane was blocked in blocking solution (PBS pH 7.5, 5% non-fat dry milk) overnight at 4 °C under mild shaking. The membrane was probed with anti-GFP mouse IgG (Roche) and Anti-Flag M2 mouse IgG (Sigma) at 1∶1000 dilution in blocking solution overnight at 4 °C, followed by 3 × 5′ wash in PBS buffer (PBS pH 7.5). The membrane was then incubated with goat anti-mouse IgG conjugated to Alkaline Phosphatase (AP) (Sigma) (1∶1000 dilution in blocking solution) for 1 h at room temperature, and rinsed 3 × 5′ with PBST. Pre-mixed NBT/BCIP AP solution (UCPH, DK) was added to the blot and incubated for color development.

### Post FACS residual gRNA activity of lysed protoplasts

20 μl of protoplasts expressing gRNA-*NbPDS2*/*Sp*Cas9 were added to 80 μl PBS, briefly vortexed and left at room temperature for 2 h; 20 μl of protoplasts were added to PBS buffer with 5 μl RNAseA/T1 (Thermo fisher #EN0551), briefly vortexed and left at room temperature for 2 h; and 20 μl of protoplasts was flash frozen with immediately addition of 80 μl PBS which were then heated for 3 min at 95 °C. Flanking primers used were P233 and P232.

### Flow cytometry and fluorescence activated cell sorting (FACS) of N. benthamiana protoplasts

The protoplast solution was first passed through 50-μm filcons (BD Biosciences) to achieve a single-cell suspension. Protoplast suspensions were cytometrically analyzed and sorted with a FACSAria III (BD Biosciences) fitted with a 100-μm nozzle and using phosphate-buffered saline (PBS) as a sheath fluid. The procedure and setting used were as described in [29] with a large nozzle size (100 μm) to provide optimal survival for most cell types and sorting based on ~ 10,000 events. Briefly, the sheath pressure was set at 20 psi, and the defection plate voltage was set at 5000 V (default “low” setting). A 488 nm Coherent Sapphire Solid State laser was used for excitation, and emission was measured at 530 nm for GFP. The photomultiplier tube voltage was set at 183 V for forward scatter, 286 V for side scatter, 308 V for GFP, and 518 V for Allophycocyanin. The threshold value for event detection was set at 8835 on forward scattering. The drop drive frequency was set to approximately 30 kHz, and the amplitude was set to approximately 45 V; the drop delay value was approximately 26 (these settings will vary slightly with day-to-day operation of the FACSAria III). Identification of viable, single protoplasts through the use of forward scatter (FSC) and side scatter (SSC) as a first gating strategy, which is routinely used for gating mammalian cells, was not attempted due to the high variability of protoplast size. Instead, the FITC and DAPI intensities were recorded as represented in dot plots. 10,000 events are displayed in each plot. Gates were set to separate and thus enable enrichment of WT and GFP transfected protoplasts, using the WT sample to define non-transfected wild type populations in the transfected samples. Transfected protoplasts were defined as FITC-positive events. Data were processed using the FACSDiva 8.0.1 software (BD Biosciences).

Viability test was done on a Sony SH800S Cell sorter, with automated setup for 130 μm microfluidics sorting chips, psi 9. PBS was used as sheath fluid, with samples sorted into flat-bottomed 96 well microtiter-plates containing 200 μl W5 buffer. For visualization purposes protoplasts were layered at the bottom of the microtiter plate by a brief centrifugation step, 100×g, 5 min.

Gating strategy on Sony SH800S cell sorter was similar to those used on the BD FACSAria III sorter.

## Additional files

Additional file 1: **Figure S1.** *Localized GFP-fluorescence of SpCas9-2A-GFP/NbPDS2-gRNA*. GFP-fluorescence of *Sp*Cas9-2A-GFP/*NbPDS2*-gRNA from *Agrobacterium* infiltrated in leaves of *N. benthamiana* 3 days post infiltration was evident in the contours epidermis cells of intact leaves (**A**). Overlay of bright field and fluorescence (FITC filter) microscopy of isolated *Sp*Cas9-2A-GFP/*NbPDS*2-gRNA transformed protoplasts regularly showed 60 - > 80% transformation efficiency (**B**). GFP fluorescence was seen in cytoplasmic strands with some nuclei accumulation (**A** and **C**), which both are in accordance with a primarily cytoplasmic localization of the GFP. (PDF 9243 kb)

Additional file 2: **Figure S2.***WT N. benthamiana protoplasts pre and post FACS*. Protoplasts were isolated as described in the Methods section, stored in buffer MMM550 on ice and immediately FACS sorted (total population sorted) into the MMM550 buffer and stored on ice. An estimated survival rate of ca 10–20% (concentric intact protoplast) was observed as evidenced by bright field (**A**) microscopy. (**B**, **C**) GFP expression analysis using confocal microscopy and viability test using propidium iodide (PI). Left panels are scan of the protoplasts expressing *Sp*Cas9-2A-GFP construct. Right panels non-transformed control. (**B**) Distinguishable GFP signal can be observed in transformed protoplasts (arrowhead). (**C**) Viability analysis using propidium iodide (PI). Non PI stained protoplast expressing GFP were observed. (PDF 7907 kb)

Additional file 3: **Figure S3.** *Post FACS residual gRNA/Cas9 activity of lysed protoplasts*. Ribonucleoprotein, i.e. in vitro transcribed gRNA mixed with heterologous expressed *Sp*Cas9 enzyme, delivered by PEG transformation, have been shown to confer efficient nuclease activity in *Arabidopsis thaliana*, tobacco, lettuce and rice protoplasts [10, 53]. We tested whether PBS mediated protoplast lysis could mediate additional extra-cellular derived indel formation resulting in an over-estimated gRNA/*Sp*Cas9 activity. Incubation 2 h at room temperature in PBS buffer resulted in a 2–3 fold increased indel formation, compared to immediate activity abolishment through flash freezing/boiling or RNAse addition, as judged by resistant RE band intensities. Lanes: Pos Ctrl (*NbPDS2*-gRNA/*Sp*Cas9 positive from leaves), Neg ctrl (WT without *NbPDS2*-gRNA/*Sp*Cas9), RT 2 h (PBS mediated lysis followed by 2 h incubation at room temperature), flash freezing (flash freezing in liquid N_2_ followed by boiling), +RNAase (RNAase addition). For experimental setup see Method section (PDF 191 kb)

Additional file 4: **Figure S4.** *GFP-fluorescent protoplasts embedded in alginate*. Single fluorescent protoplasts are visible as evidenced by fluorescent (FITC filter) microscopy before (**A**) and after alginate embedment (**B**). Calli formation (**C**) of a single protoplast as evidenced by bright field microscopy. Protoplast embedment in alginate is described in the Methods section. (PDF 4381 kb)

Additional file 5: **Table S1.** *Vector construct and primer list (DOCX 18 kb)*

## Data Availability

All constructs used in the present study are listed in Additional file 5: Table S1 and will be available upon request. Basic vector constructs (51491, 51493, 51494, 52255) were from and are available from Addgene (https://www.addgene.org/).

## Abbreviations

*A. tumefaciens*: *Agrobacterium tumefaciens*
*AtU6*-Pro: *Arabidopsis thaliana U*6 promoter
*BeYDV*: Bean yellow dwarf virus
CMV35S: Cauliflower mosaic virus 35S promoter
CRISPR-Cas: Clustered Regularly Interspaced Short Palindromic Repeats (CRISPR)/CRISPR-associated systems (Cas))
FACS: Fluorescence Activated Cell Sorting
FMDV, 2A: Self-cleaving sequence of foot-and-mouth disease virus, 2A
gRNA: guide RNA
GVR: Gemini Virus Replicon
HPT: Hygromycin phosphotransferase
HR: Homologous Recombination
IDAA: Indel Detection by Amplicon Analysis
indels: Deletions and insertions
LB & RB: Left and Right T-DNA border
*N. benthamiana*: *Nicotiana benthamiana*
*NbPDS*: Phytoene desaturase
*NbRRA*: REDUCED RESIDUAL ARABINOSE arabinosyl transferase
NHEJ: Non-Homologous End Joining
NLS: Nucleus Localisation Signal
NOS: Nopaline synthase terminator
PAM: Protospacer adjacent motif
PGE: Precise genome editing
RE: Restriction Enzyme
*Sp*Cas9: *Streptococcus pyogenes* Cas9
TALENs: Transcription activator-like effector nucleases
ZFNs: Zinc Finger-Nucleases
