# Supplementary figures and images for: Improved CRISPR/Cas9 gene editing by fluorescence activated cell sorting of green fluorescence protein tagged protoplasts
Source: BMC Biotechnol. 2019 Jun 17;19:36. doi: 10.1186/s12896-019-0530-x (PMC6580576; doi:10.1186/s12896-019-0530-x)

A. <sup>35S</sup> *F-SpCas9* 2A GFP <sup>NOS</sup> <sup>U6</sup> *NbPDS-CRISPR* <sup>TT</sup>

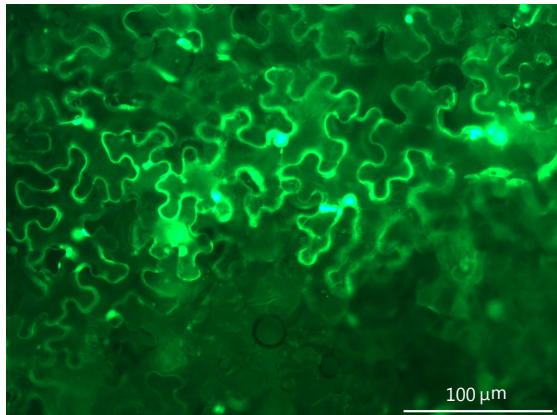

B.

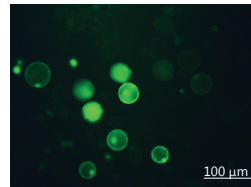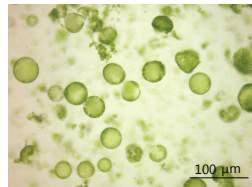

C.

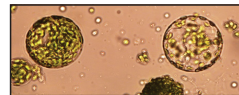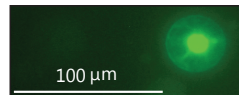

Supplement: Supplementary file 1 — Figure S1. Localized GFP-fluorescence of SpCas9-2A-GFP/NbPDS2-gRNA. GFP-fluorescence of SpCas9-2A-GFP/NbPDS2-gRNA from Agrobacterium infiltrated in leaves of N. benthamiana 3 days post infiltration was evident in the contours epidermis cells of intact leaves (A). Overlay of bright field and fluorescence (FITC filter) microscopy of isolated SpCas9-2A-GFP/NbPDS2-gRNA transformed protoplasts regularly showed 60 - > 80% transformation efficiency (B). GFP fluorescence was seen in cytoplasmic strands with some nuclei accumulation (A and C), which both are in accordance with a primarily cytoplasmic localization of the GFP. (PDF 9243 kb) [file 12896_2019_530_MOESM1_ESM.pdf]

Petersen et al; Supplementary Figure 3

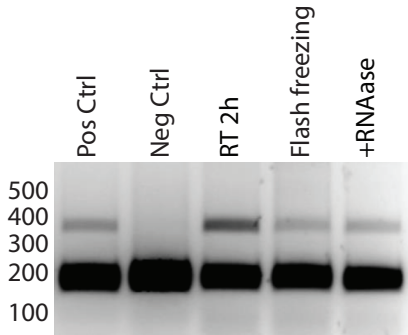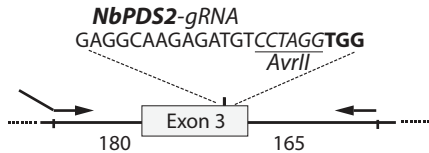

Supplement: Supplementary file 3 — Figure S3. Post FACS residual gRNA/Cas9 activity of lysed protoplasts. Ribonucleoprotein, i.e. in vitro transcribed gRNA mixed with heterologous expressed SpCas9 enzyme, delivered by PEG transformation, have been shown to confer efficient nuclease activity in Arabidopsis thaliana, tobacco, lettuce and rice protoplasts [10, 53]. We tested whether PBS mediated protoplast lysis could mediate additional extra-cellular derived indel formation resulting in an over-estimated gRNA/SpCas9 activity. Incubation 2 h at room temperature in PBS buffer resulted in a 2–3 fold increased indel formation, compared to immediate activity abolishment through flash freezing/boiling or RNAse addition, as judged by resistant RE band intensities. Lanes: Pos Ctrl (NbPDS2-gRNA/SpCas9 positive from leaves), Neg ctrl (WT without NbPDS2-gRNA/SpCas9), RT 2 h (PBS mediated lysis followed by 2 h incubation at room temperature), flash freezing (flash freezing in liquid N2 followed by boiling), +RNAase (RNAase addition). For experimental setup see Method section (PDF 191 kb) [file 12896_2019_530_MOESM3_ESM.pdf]

A

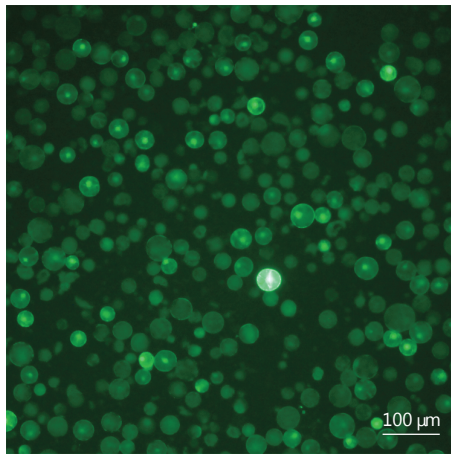

Pre-alginate embedding

B

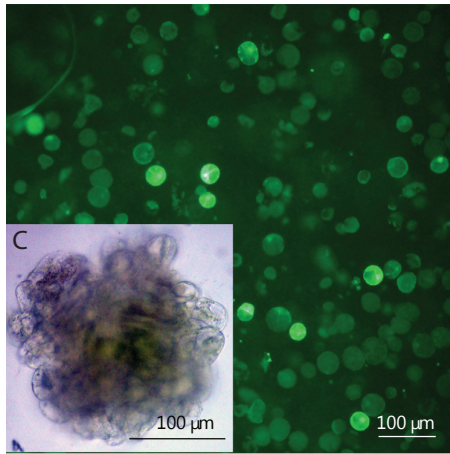

Alginate embedded

<sup>35S</sup> GFP NOS

Supplement: Supplementary file 4 — Figure S4. GFP-fluorescent protoplasts embedded in alginate. Single fluorescent protoplasts are visible as evidenced by fluorescent (FITC filter) microscopy before (A) and after alginate embedment (B). Calli formation (C) of a single protoplast as evidenced by bright field microscopy. Protoplast embedment in alginate is described in the Methods section. (PDF 4381 kb) [file 12896_2019_530_MOESM4_ESM.pdf]
