# Supplementary material for: Improved CRISPR/Cas9 gene editing by fluorescence activated cell sorting of green fluorescence protein tagged protoplasts
Source: BMC Biotechnol. 2019 Jun 17;19:36. doi: 10.1186/s12896-019-0530-x (PMC6580576; doi:10.1186/s12896-019-0530-x)
Supplement: Supplementary file 2 — Figure S2.WT N. benthamiana protoplasts pre and post FACS. Protoplasts were isolated as described in the Methods section, stored in buffer MMM550 on ice and immediately FACS sorted (total population sorted) into the MMM550 buffer and stored on ice. An estimated survival rate of ca 10–20% (concentric intact protoplast) was observed as evidenced by bright field (A) microscopy. (B, C) GFP expression analysis using confocal microscopy and viability test using propidium iodide (PI). Left panels are scan of the protoplasts expressing SpCas9-2A-GFP construct. Right panels non-transformed control. (B) Distinguishable GFP signal can be observed in transformed protoplasts (arrowhead). (C) Viability analysis using propidium iodide (PI). Non PI stained protoplast expressing GFP were observed. (PDF 7907 kb) [file 12896_2019_530_MOESM2_ESM.pdf]

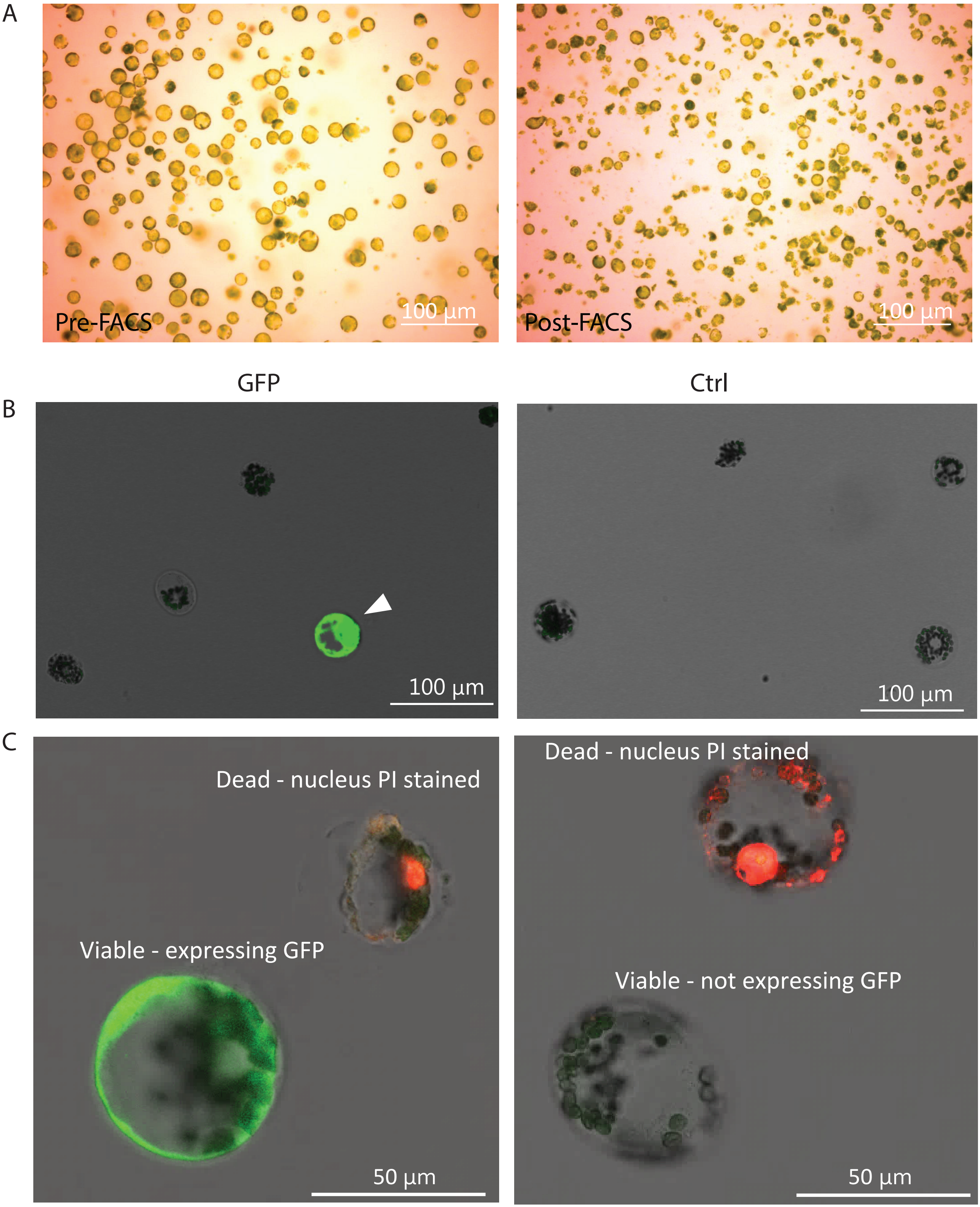
A
Pre-FACS
100 µm
Post-FACS
100 µm
GFP
Ctrl
B
100 µm
100 µm
C
Dead - nucleus PI stained
Viable - expressing GFP
50 µm
Dead - nucleus PI stained
Viable - not expressing GFP
50 µm
