# Supplementary material for: Improved CRISPR/Cas9 gene editing by fluorescence activated cell sorting of green fluorescence protein tagged protoplasts
Source: BMC Biotechnol. 2019 Jun 17;19:36. doi: 10.1186/s12896-019-0530-x (PMC6580576; doi:10.1186/s12896-019-0530-x)
Supplement: Supplementary file 5 — Table S1. Vector construct and primer list (DOCX 18 kb) [file 12896_2019_530_MOESM5_ESM.docx]

**Supplementary Table 1** - *Vector construct and primer list*

| M001 | pUC57_*Sp*Cas9-2A-mTur-Nos |
| --- | --- |
| V152 | AttL1-*Sp*Cas9-2A-mTur-Nos; *AtU6*-*NbRRA*all1-gRNA-TT-AttL2 |
| V82 | pLSLGFP-R_v2 |
| V126 | pREP |
| V153 | AttL1-*Sp*Cas9-2A-mTur-Nos; *AtU6*-*NbPDS2*-gRNA-TT-AttL2 |
| V197 | pUC57_AttL1-*Sp*Cas9-2A-GFP-Nos; *AtU6*-*NbRRA*all1-gRNA-TT-AttL2 |
| V198 | pUC57_AttL1-*Sp*Cas9-2A-GFP-Nos; *AtU6*-*NbPDS2*-gRNA-TT-AttL2 |
| V199 | pLSL_V2_ LIR-AttB1-*Sp*Cas9-2A-GFP-Nos; *AtU6*-*NbRRA*all1-gRNA-TT-AttB2 SIR-35S-LIR |
| V200 | pLSL_V2_LIR-AttB1-*Sp*Cas9-2A-GFP-Nos; *AtU6*-*NbPDS2*-gRNA-TT-AttB2 SIR-35S-LIR |
| pREP | pMDC32_35S-Rep/RepA-Nos |
| pLSL_V2 | pCambia 1300_LIR-AttR1-ccdb-CmR-AttR2 SIR-35S-LIR |
| V26 | pUC57_attL1-*AtU6*:*BbsI*-*BbsI*-tracr-TT_AttL2 |
| V207 | pUC57_attL1-*AtU6*:*NbPDS*-tracr-TT_AttL2 |
| V208 | pUC57_attL1-*AtU6*:*NbRRA*all1-tracr-TT_AttL2 |

*Primer list* (Bold designates FAM primer overhang)

| P348_*NbPDS*5F | CTGAGCCATGATAAGCGGGT |
| --- | --- |
| P230_*NbRRA*all1-F | AACGCCACAACCAGATAATTGG |
| P231_*NbRRA*all1-R | **AGCTGACCGGCAGCAAAATT**GCGTTGGACTTGATATAAAATGCTTG |
| P232_*NbPDS2*R | CACCCTTGCAATTGTTTGAGC |
| P233_*NbPDS2*_F | **AGCTGACCGGCAGCAAAATTG** CTAGCCGAGGTACTACATGG |
| P342_*NbRRA*5R | ATCCAACGCCACAACCAGAT |
| P346_*NbRRA*F7 | CCTAATGGATTCTTCTCTCCCG |
| P319 *NbPDS2*F | **AGCTGACCGGCAGCAAAATTG**GCTTGCATAGTACTGTGCC |
| P320*NbPDS2*R | GACTACCAATAGGCAAGGACAC |
| P321 *NbRRA*2F | CGTTGGACTTGATATAAAATGCTTG |
| p322 *NbRRA*2R | **AGCTGACCGGCAGCAAAATTG**CAGCCACCTGAAGCTTTTG |
| PFAMF | AGCTGACCGGCAGCAAAATTG |
| P230_*NbRRA*R | AACGCCACAACCAGATAATTGG |
| P231_*NbRRA*allF | **AGCTGACCGGCAGCAAAATTG**CGTTGGACTTGATATAAAATGCTTG |
| P233_*NbPDS2*F | **AGCTGACCGGCAGCAAAATTG**CTAGCCGAGGTACTACATGG |
| P212 | GGCTACTAGTGAATTACCATGGATTACAAGGATGATGATGATAAG |
| P077 | GACGTCACCGCATGTTAGCAGACTTCCTCTGCCCTCTCCACTGCC  CTTCTTCTTCTTAGCCTGTCCAG |
| L1 | ACATGCGGTGACGTCGAGGAGAATCCTGGCCCACGATCGAGATCTATGGTGTCGAAGGGCGAGGAGC |
| L2 | GCGTTTAAACGAATTTCTAGAGCGGCCGCGATCTAGTAACATAGATGA |
| P042 | gattgCCAATTGTGAATCCACGGAG |
| P043 | aaacCTCCGTGGATTCACAATTGGc |
| P149 | GATT GAGGCAAGAGATGTCCTAGG |
| P150 | AAAC CCTAGGACATCTCTTGCCTC |
